# Preclinical Pharmacokinetics and Dosimetry of an ^89^Zr Labelled Anti-PDL1 in an Orthotopic Lung Cancer Murine Model

**DOI:** 10.3389/fmed.2021.741855

**Published:** 2022-01-31

**Authors:** Anis Krache, Charlotte Fontan, Carine Pestourie, Manuel Bardiès, Yann Bouvet, Pierre Payoux, Etienne Chatelut, Melanie White-Koning, Anne-Sophie Salabert

**Affiliations:** ^1^CRCT, UMR 1037, Université de Toulouse, INSERM, Université Paul-Sabatier, Toulouse, France; ^2^ToNIC, Toulouse NeuroImaging Center, UMR 1214, Université de Toulouse, INSERM, Université Paul-Sabatier, Toulouse, France; ^3^General-Electric - Zionexa, Targeting Imaging and Therapy, Buc, France; ^4^CREFRE (Centre Régional D'Exploration Fonctionnelle et Ressources Expérimentales) – INSERM UMS006, Plateforme GénoToul-Anexplo, Toulouse, France; ^5^ENVT (Ecole Nationale Vétérinaire de Toulouse), Toulouse, France; ^6^IRCM (Institut de Recherche en Cancérologie de Montpellier), UMR 1194 INSERM, Université de Montpellier and ICM, Montpellier, France; ^7^Département de Médecine Nucléaire, ICM (Institut du Cancer de Montpellier), Montpellier, France; ^8^Centre Hospitalo-Universitaire de Toulouse, Toulouse, France

**Keywords:** anti-PDL1, zirconium-89, PET, dosimetry, pharmacokinetics, preclinical

## Abstract

Anti-PDL1 is a monoclonal antibody targeting the programmed death-cell ligand (PD-L1) by blocking the programmed death-cell (PD-1)/PD-L1 axis. It restores the immune system response in several tumours, such as non-small cell lung cancer (NSCLC). Anti-PDL1 or anti-PD1 treatments rely on PD-L1 tumoural expression assessed by immunohistochemistry on biopsy tissue. However, depending on the biopsy extraction site, PD-L1 expression can vary greatly. Non-invasive imaging enables whole-body mapping of PD-L1 sites and could improve the assessment of tumoural PD-L1 expression.

**Methods:**

Pharmacokinetics (PK), biodistribution and dosimetry of a murine anti-PDL1 radiolabelled with zirconium-89, were evaluated in both healthy mice and immunocompetent mice with lung cancer. Preclinical PET (μPET) imaging was used to analyse [^89^Zr]DFO-Anti-PDL1 distribution in both groups of mice. Non-compartmental (NCA) and compartmental (CA) PK analyses were performed in order to describe PK parameters and assess area under the concentration-time curve (AUC) for dosimetry evaluation in humans.

**Results:**

Organ distribution was correctly estimated using PK modelling in both healthy mice and mice with lung cancer. Tumoural uptake occurred within 24 h post-injection of [^89^Zr]DFO-Anti-PDL1, and the best imaging time was at 48 h according to the signal-to-noise ratio (SNR) and image quality. An *in vivo* blocking study confirmed that [^89^Zr]DFO-anti-PDL1 specifically targeted PD-L1 in CMT167 lung tumours in mice. AUC in organs was estimated using a 1-compartment PK model and extrapolated to human (using allometric scaling) in order to estimate the radiation exposure in human. Human-estimated effective dose was 131 μSv/MBq.

**Conclusion:**

The predicted dosimetry was similar or lower than other antibodies radiolabelled with zirconium-89 for immunoPET imaging.

## Introduction

Lung cancer is the most common cause of death from cancer in the world with an estimated incidence of 2.09 million new cases and 1.76 million deaths in 2018 (1, 2). There are two histological types of lung cancer in humans: non-small cell lung cancer (NSCLC) in 80% of patients and small cell lung cancer in the remaining 20% patients (3–5). In 70% of cases, lung cancer is diagnosed at an advanced stage (6). The chances of overall survival at 5 y are considerably reduced depending on the stage of detection, ranging from 67% at stage I to 1% at stage IV (7). New classes of immunotherapy based on immune checkpoint inhibitors targeting programmed cell-death ligand 1 (PD-L1) and programmed cell death 1 (PD-1) have been shown to be efficient in NSCLC. Indeed, in lung cancers, tumour cells can overexpress PD-L1, which binds to PD-1 on cytotoxic T cells (CTL). This interaction blocks the CTL effector signal, limiting immune response, and enabling tumoural growth and invasion. Immune checkpoint inhibitors of the PD-1/PD-L1 axis (nivolumab, pembrolizumab, or atezolizumab) represent a major advance in lung cancer treatment and have improved the overall survival of patients with NSCLC. However, response rates for these treatments do not exceed 45% in first-line and 30% in second-line treatment (8–11). Moreover, resistance phenomena or hyper-progression under these treatments is increasingly described (12). Identifying patients who could benefit from these therapies remains a challenge (13). Immunohistochemistry tests on tumoural biopsy tissue, such as SP142 for atezolizumab (anti-PDL1) or 28-8 for nivolumab (anti-PD1), have been approved by health regulation authorities to determine PD-L1 expression prior to treatment to predict benefit for patients (14, 15). However, some patients with low PD-L1 scores also show response under anti-PDL1 immunotherapy (16, 17). Biopsy samples may present false negatives related to intra-tumoural heterogeneity of PD-L1 expression and heterogeneity between the primary and metastatic tumour sites (14, 18).

PET is a functional and non-invasive imaging modality based on the use of radiolabelled molecules (such as radiolabelled antibodies and metabolic tracers) targeting biomarkers. This imaging technique can be used to assess tumoural PD-L1 expression without being limited by the sampling issues involved in biopsy and help the clinician to decide whether a patient is eligible for anti-PD1 or anti-PDL1 treatment (19–21). Immuno-PET (using total or specific section of the radiolabelled antibody) could benefit from the specificity and selectivity of such molecules. The most common PET isotope in clinical diagnosis is the fluorine 18 [^18^F] with a half-life of 109.7 min but it is not suitable for antibodies with long biological half-lives (22). However, other more appropriate radioisotopes, such as zirconium-89 ([^89^Zr]), with a physical half-life of 78.4 h can be used (23). [^89^Zr] decay proceeds *via* positron emission (23%, *E*_β*max*_ = 902 keV) and electron capture (77%). The two main γ radiation emissions are the 511 keV from positron annihilation and the 909 keV from the transition of ^89^mY to ^89^Y (24, 25).

The aim of the study was to evaluate the biodistribution of murine anti-PDL1 radiolabelled with [^89^Zr] in healthy and lung cancer-grafted mice. The area under the concentration-time curve (AUC) was estimated using pharmacokinetic (PK) modelling in healthy mice organs. Allometric scaling (26) was then used to estimate PK parameters in human (from the murine experimental data) and to calculate both human organ dose exposure and human effective dose.

## Materials and Methods

All chemical compounds were purchased from Sigma-Aldrich (Saint-Louis, MI, USA) except where otherwise specified.

### Radiosynthesis of [^89^Zr]DFO-Anti-PDL1

The radiolabelling procedure was based on Vosjan et al. (27). (1) Chelation: Between 5 and 5.5 mg of anti-PDL1 (Bio X Cell Cat# BE0101, RRID:AB_10949073, clone 10F.9G2, West Lebanon) in a 750 μl of phosphate-saline buffer (PSB) were mixed with 150–200 μl of 0.1 M sodium carbonate to buffer the solution at a pH between 8.5 and 9. A concentration of 40 nM of p-NCS-Bz-DFO (Macrocyclics, Plano, TX, USA) diluted in DMSO was added to the antibody solution and incubated at 37°C for 30–45 min. The chelated antibodies were purified by elution with 5 mg/ml gentisic acid and 0.25 M sodium acetate solution on PD-10 size exclusion (GE Healthcare, Boston, MA, USA). (2) Radiolabelling: In total, 200 μl of oxalic acid 1 M were added to 37 MBq of [^89^Zr] oxalate solution (20 μl; Perkin-Helmer, Waltham, Massachusetts, USA), followed by 90 μl of sodium carbonate 2 M solution. After waiting 3 min, 1 ml of 0.5 M hydroxyethyl piperazineethanesulfonic acid (HEPES) followed by 710 μl of chelated antibody solution was added. After 90 min incubation at 37°C, the solution of p-NCS-Bz-DFO radiolabelled antibody was purified using the PD-10 column. Radiochemical purity was performed by radio-chromatogram (LabLogic, UK) using Thin-Layer Chromatography (TLC) strips (Biodex, Shirley, NY, USA) with citric acid at 20 mM and sodium carbonate at 0.1 M solution as mobile phase. Phosphate buffer with 150 mM NaCl with a 1 ml/min flow rate and size exclusion chromatography column were used for the analytical procedure (Bio-SEC A-300, Agilent, Santa Clara, CA, USA). For the *in vitro* stability assay, [^89^Zr]DFO-anti-PDL1 was incubated at 37°C in human plasma and at 4°C in PSB. Radio-HPLC was performed at 24 and 168 h in the plasma and at 24 and 168 h in the PSB to quantify the proportion of free [^89^Zr] and the [^89^Zr]DFO-anti-PDL1.

### Cell Culture

CMT167 (ECACC Cat# 10032302, RRID: CVCL_2405) cells were used to induce the lung cancer. CMT167 cells are highly metastatic murine lung cancer cells provided by Sigma-Aldrich and known to express PD-L1 protein on their surface (28–30). The cells were maintained using culture Eagle's minimal essential medium (DMEM) and 10% Foetal bovine serum (FBS) in T75 flasks. They were incubated at 37°C and 5% CO_2_. In the binding and immunoreactivity (IR) experiments, cells in the growth phase (confluence > 75%) were incubated in 6-well plates the day before the experiment.

### Binding and IR Assay

The binding and IR experiments are based on the method of Lindmo et al. (31). For the *binding assay*, CMT167 cells 1.5 × 10^6^ per well in 2 ml of DMEM were rinsed twice with 1 ml 1% BSA/PBS before incubating with [^89^Zr]DFO-anti-PDL1. The concentrations used for total labelling ranged from 2.5 to 100 nM in 1% BSA/PBS solutions. For non-specific labelling, an excess of 200-fold higher native antibody was added. After 2 h incubation at 37°C and slight agitation, cells were rinsed 3 times with 1% BSA/PBS solution and then recovered with trypsin/ethylenediaminetetraacetic acid (EDTA; 1 ml and 10 min incubation). To calculate the affinity constant (K_D_), we used a non-linear fitting curve on GraphPad with the module One *Site—specific binding*.

For the *IR assay*, we used CMT 167 with a cell concentration ranging from 0.125 × 10^6^ cells to 1.5 × 10^6^ cells per well and an antibody concentration of 2 nM for total binding. For non-specific *IR*, a 200-fold higher native antibody concentration was added. After 2 h incubation at 37°C and slight agitation, cells were rinsed 3 times with 1% PBS/BSA solution and then recovered with trypsin/EDTA (1 ml and 10 min incubation time). Counting of trypsin cells was performed using a gamma counter (Perkin Elmer, Waltham, MA, USA) for 15 min per tube.

### Subjects/Experimental Animals

This study was conducted under acceptance of the protocol by the Ethics Committee (no. 22816-2019111216307851). Fourteen C57BL/6 mice (IMSR Cat# JAX:000664, RRID:IMSR_JAX:000664) aged 6–10 wk and an average weight of 25 g were used. The tumour model was developed according to Li et al. (29) by injecting intrapulmonary 10,000–15,000 CMT167 cells through the left thoracic cage under 3% isoflurane.

### Immunohistochemical Analysis of PD-L1

Ten days after lung cancer induction, mice were euthanized by cervical dislocation and lungs were harvested. Lungs (with tumour) were perfused with formaldehyde (37%) and embedded in paraffin. Specimens were sectioned at 5 μm and were dewaxed by heating for 10 min and using xylene. Sections were rehydrated using ethanol (100%). The sections were then incubated with a primary antibody, anti-PDL1 (PA5-20343, ThermoFisher, Waltham, MA, USA) at 1:200 dilution (overnight) and revealed by DAB Kit (3,3′-Diaminobenzidine, Vector, Olean, NY, USA). Haematoxylin coloration was added for nucleus coloration. Histological sections were scanned using a Panoramic 250 slide scanner (3D HISTEC, Hungary).

### Image Acquisition

After 1 wk of tumoural development, the mice were anaesthetized with isoflurane (4% for induction reduced to 2.5%) and intravenous (IV) injection (caudal or retro orbital) of [^89^Zr]DFO-anti-PDL1 was performed. For μTEP camera acquisition, the mice were kept under 2.5% isoflurane. The images were acquired using NanoScan PET/CT (MEDISO, Hungary). CT acquisitions were performed during 10 min (parameters: 35 kVp, 800 μA) followed by whole-body static PET images (energy window: 400–600 KeV), during 30 min, in list mode and reconstructed using 3D mode (Tetra-Tomo3D Mediso) with 4 iterations and 6 subsets. The size of the images initially reconstructed (mm) was 406 × 406 × 377 (*x, y, z*). The voxel size (μm) was 251 × 251 × 251. Static PET imaging acquisition (30 min) of the mice was performed at several timepoints after injection of the [^89^Zr]DFO-anti-PDL1 from day 1 to day 7. An attenuation correction, scatter correction, decay correction, and random correction were used for PET acquisition. Four acquisitions were performed over 1 wk at 24, 48, 72, and 168 h after injection of [^89^Zr]DFO-anti-PDL1.

### *Ex vivo* Biodistribution Study

Lung cancer-grafted mice with blocking (*n* = 2) received a 500 μg cold dose of anti-PDL1 at the same time as tracer IV injection ([^89^Zr]DFO-anti-PDL1). Lung cancer tumoural non-blocking mice (*n* = 4) and healthy non-blocking mice (*n* = 7) received an IV injection of [^89^Zr]DFO-anti-PDL1 alone. Animals were euthanized by cervical dislocation on day 7, and organs and the tumour were harvested, weighed, and counted in a gamma counter (for 1 min) for [^89^Zr] activity (Hidex Automatic Gamma Counter, LabLogic). These data were decay-corrected at the time of injection and background subtracted. The percentage of injected dose per gramme (%ID/g) for each organ was computed by dividing these corrected data by the activity injected.

### Image Processing

Image processing was carried out with PMOD 3.9 software (LLC technology, Switzerland, RRID:SCR_016547). PET and CT DICOM format were co-registered using the Pfuse module using rigid matching. Five organs of interest were identified (heart, lungs, kidneys, liver, and bone) using five distinct volumes of interest (VOI) per organ of 1 mm^3^, non-overlapping, and with a minimum distance of 1 mm between them (except for the bone where 3 VOI were considered). The remaining activity in the body was calculated by subtracting organ activity from the total body activity. This quantification method was inspired by Vicente et al. (32). The radioactive concentration in each organ was the average of the radioactive concentration in all the VOIs (5 per organ, in MBq per cm^3^ given by PMOD 3.9). Henceforth, we will refer to this method as the partial method.

To validate the partial method, we compared it to the whole organ contouring method obtained with PET-CT images for the heart (*n* = 6) and the liver (*n* = 6). To assess the homogeneity of the organs, we extracted the average radioactive value per voxel and the coefficient of variation (CV) using “average statistic” in PMOD VOI statistic.

The bone VOI was drawn from femur epiphysis using 3 VOIs. This region was chosen on purpose to overestimate bone dosimetry. The tumour VOI was defined using the partial method. The number of VOI depends on tumour size (VOIs between 1 and 3).

More details are given in the Supplementary Figure 1 (PET method validation in the healthy group), Supplementary Figure 2 (tumoural quantification using the partial method), Supplementary Table 5 (detail of calculations for estimating the organ concentration), and Supplementary Table 6 (final comparison and validation of the partial method).

### Blood Samples

In order to evaluate the PK of [^89^Zr]DFO-anti-PDL1, blood samples of 50 μl from the cheek vein were taken at the following times after imaging: 1, 3, 24, 48, 72, and 168 h. The samples were weighed and counted with a gamma counter (Hidex Automatic Gamma Counter, LabLogic). Radioactive decay was automatically corrected by the software related to the injection time.

### PK Analyses

Non-compartmental (NCA) and compartmental (CA) PK analyses were performed using Monolix/PKanalix software (Lixoft, France). For the NCA, the PK parameters were obtained using the log-linear trapezoidal method.

Non-compartmental PK analysis is a robust method for estimating the AUC using observed data. However, considering the lack of concentration data in order to estimate the terminal slope in some organs, the elimination phase may not be correctly described using this method. Therefore, we also used population pharmacokinetic (POPPK) modelling, which is a CA method where the estimated values of AUC in each organ are based on population distributions and thus take into account inter-individual variability.

Pharmacokinetic analyses were carried out for two purposes.

#### Blood

Non-compartmental analysis was performed on blood samples in both healthy mice and mice with lung cancer to estimate and compare PK parameters.

For CA, 2-CMT models were tested with a first-order elimination (k) from the central compartment, a volume of distribution of the central compartment (V), a second compartment with intercompartmental clearance (Q), its own volume of distribution (V2), and bolus administration to describe blood concentration of [^89^Zr]DFO-anti-PDL1. Different error models were tested: constant, proportional, and combined. Model selection was based on goodness-of-fit plots, model stability, and relative standard error (RSE%) of the estimated parameters.

#### Organs

We performed NCA to obtain the AUC_0to168h_ in each organ, and these values were compared in healthy and lung cancer-grafted mice to assess biodistribution of [^89^Zr]DFO-anti-PDL1. A 1-CMT (CA) model with a first-order absorption (ka), a first-order elimination (ke), and the volume of distribution (V) was used to describe the time-activity curve for each organ and to obtain an AUC for both AUC_0to168h_ and AUC_0toinfinity_. Before mouse to human extrapolation, the robustness of the 1-CMT organ model was tested by calculating the root mean square error (RMSE) between the CA AUC_0to168h_ and the NCA AUC_0to168h_. From a PK perspective, the current model will capture the radioactive concentration within the organs and not the distribution of [^89^Zr]DFO-anti-PDL1 (examples of curve fitting and AUC estimation are shown in Supplementary Figure 4).

### Dosimetry

Reference dosimetry is required for documenting the irradiation delivered by new radiopharmaceuticals before asking for institutional drug approval. According to the International Commission on Radiological Protection (ICRP) recommendations, PK parameters should be entered in anthropomorphic reference models. Reference models (e.g., ICRP 110 models in our study) provide a reference geometry, such as a variety of organs with a realistic shape, disposition, and composition. Sources (i.e., regions where activity can be measured, for example, by quantitative imaging) are accounted for to provide the irradiation to all organs of the reference model. Results are therefore presented for all target organs/tissues that can be irradiated from the source organs/tissues where the radiopharmaceutical is uptaken (33).

[^89^Zr]DFO-anti-PDL1 concentrations in each organ were estimated with no decay-activity correction by the 1-CMT PK model for the AUC_0toinfinity_. We extrapolated the PK from mouse to human using the McParland equation (34). Total activity in the rest of the body was calculated by removing the sum of mean residence time (MRT) in the five organs from the whole-body MRT in the mouse. Then the MRT in the mouse was extrapolated to obtain values in human.


$$\begin{array}{l} {\;\tilde{\text{A}}\;}_{organ,\;Human\;}={\left(\frac{m_{Animal}}{m_{Human}}\right)}_{WB}\times \;{\left(\frac{m_{Human}}{m_{Animal}}\right)}_{Org}\times \;{\;\tilde{\text{A}}\;}_{Organ,Animal} \end{array}$$

Where, WB is the whole body, Org is the organ, Ã is cumulated activity (kBq.h), and m is the mass in kg.

Thus, extrapolated human AUC _0toinfinity_ was used to estimate organ radiation exposure and effective dose using IDAC-Dose 2.1 software for adult men and according to ICRP 103 (35). More details regarding calculations are provided as Supplementary Data.

### Statistics

For comparisons of quantitative variable distributions, non-parametric Wilcoxon-Mann-Whitney or Kruskal-Wallis tests were used (5% significance level) with R version 3.6.1 (RRID:SCR_000432). Figures were generated using GraphPad Prism version 8.3 (RRID:SCR_002798), Monolix version 2020 (Simulations Plus, RRID:SCR_003946), and PMOD 3.9.

## Results

### Radiolabelling, Binding, and IR

Radiolabelling yield (*n* = 7) was 46 ± 13% for all radiolabelling assays. The best radiolabelled [^89^Zr]DFO-anti-PDL1 solutions were selected for *in vitro* and *in vivo* studies (*n* = 3). In this subset, we obtained an radiochemical purity (RCP) of 2.2 GBq/μmol (±0.15 GBq/μmol), a volumetric activity of 6.6 MBq/ml (±0.86 MBq/ml), and a concentration of 0.45 mg/ml (±0.06 mg/ml).

**Figure 1:**
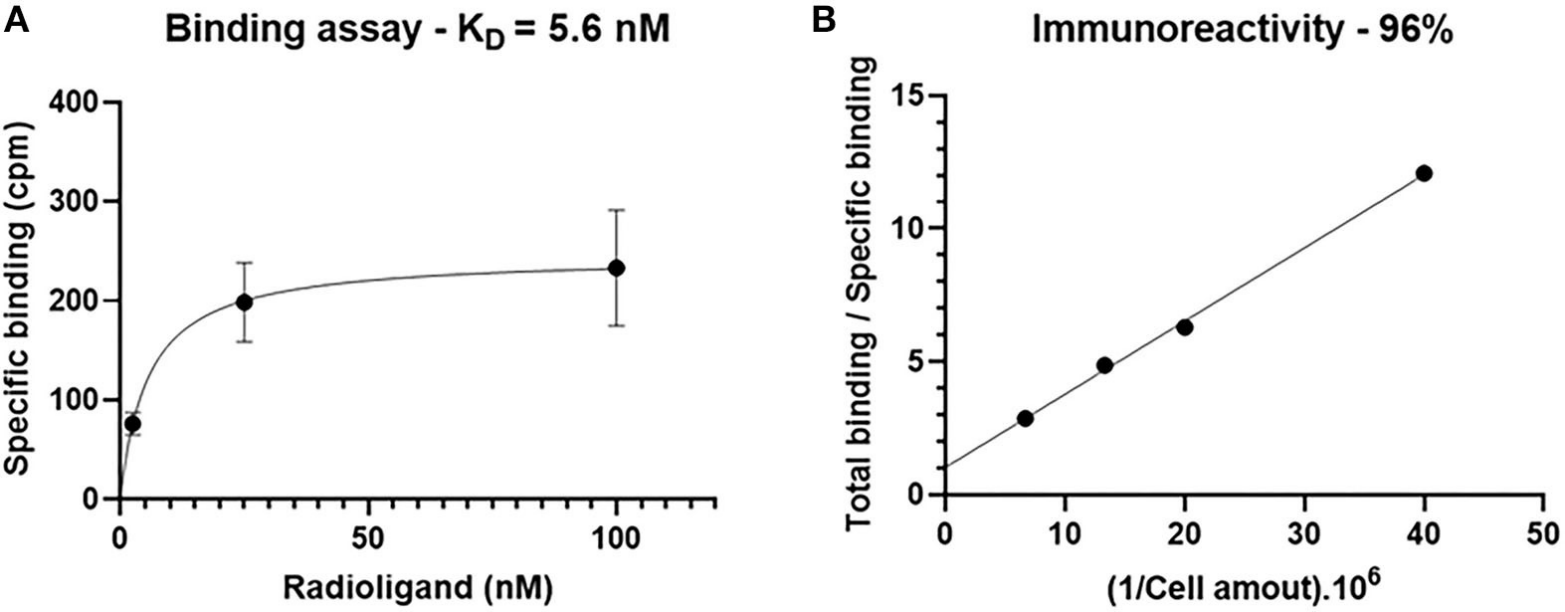
Binding and immunoreactivity assays. **(A)** Binding assay was performed by subtracting total binding and non-specific binding by using constant cell concentration and increasing [^89^Zr]DFO-anti-PDL1 concentration, Kd = 5.6 nM. Error bars represent standard errors. **(B)** Immunoreactivity was performed using an increasing cell concentration and constant [^89^Zr]DFO-anti-PDL1 concentration (2 nM). Y intercept indicates the immunoreactive fraction, 96%.

*In vitro* tests were performed on CMT167 cells expressing PD-L1 protein. An affinity constant (Kd) of 5.6 nM (*R*^2^ = 0.99) and an IR of 96% (*R*^2^ = 0.99) were found (Figure 1). PSB *in vitro* stability assay revealed that the [^89^Zr]DFO-anti-PDL1 was stable up to 168 h with a radiochemical purity superior to 95%. RCP in plasma *in vitro* stability assay was superior to 95% at 24 h and decreased to 75% at 168 h.

### Validation of PET Method

On 6 healthy mice, the liver and the heart were used to validate the PET quantification using the partial contouring method and whole-body contouring method. We found that the concentration in the organ was homogenous and comparable to the whole organ contouring method with no significant differences between both methods (*p* = 0.48 for the liver and *p* = 0.86 for the heart, Wilcoxon test). We found a CV of 22% whereas the partial method gave a CV of 20%, suggesting that there the variation of the radioactivity in the organs is similar whichever contouring method is used.

### PK Analyses of [^89^Zr]DFO-Anti-PDL1

In this study, the average radiolabelled antibody dose injected per mouse was about 1.9 mg/kg, and NCA was used to compare the biodistribution of [^89^Zr]DFO-anti-PDL1 in blood and organs in both healthy and lung cancer-grafted mice.

#### Histological Examination of PD-L1

The programmed death-cell ligand expression is assessed by immunohistochemistry on histological slides as can be seen in **Figures 4A,B**. In **Figure 4A**, we can distinguish the intensity of the PD-L1 expression between the healthy lung (blue arrow) and in the tumoural cells (black arrows). The tumoural cells are discriminated by cell density. PD-L1 expression is well defined on the cell contours as shown in **Figure 4B**. These results demonstrate that 10 days after tumoural induction, CMT167 cells express PD-L1.

#### *In vivo* PET-CT Biodistribution in Healthy Mice

**Figure 2:**
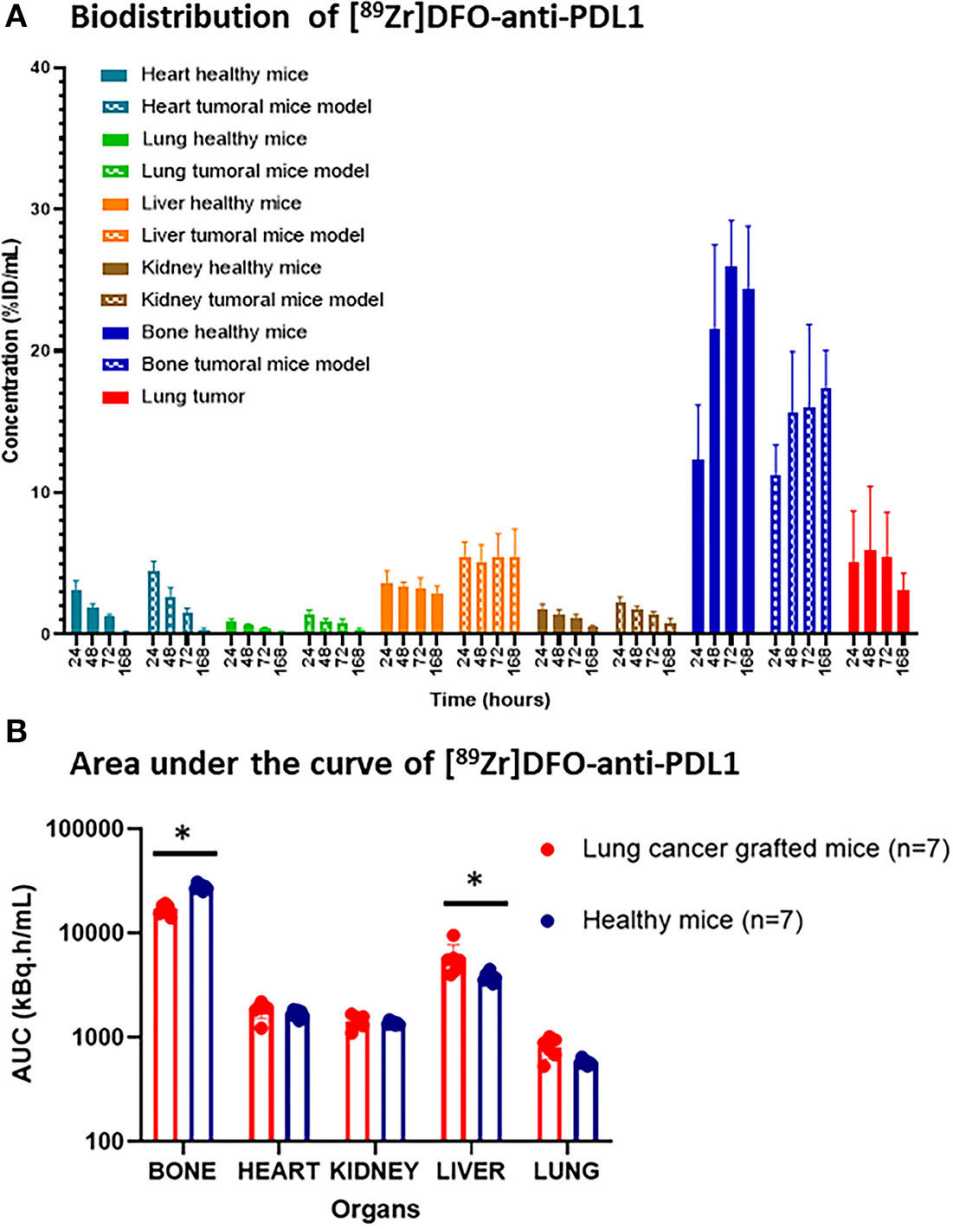
Biodistribution and area under the concentration curve of [^89^Zr]DFO-anti-PDL1 in healthy and lung cancer-grafted mice. **(A)** Biodistribution of [^89^Zr]DFO-anti-PDL1 in both healthy and lung cancer-grafted mice. **(B)** Area under the concentration curve up to 168 h after injection (AUC 0–168 h) for each organ in healthy and lung cancer-grafted mice. Wilcoxon-Mann-Whitney test (*p < 0.05). AUC, the area under the curve.

Seven mice with an average weight of 25 ± 0.7 g were IV injected with [^89^Zr]DFO-Anti-PDL1 (742 ± 38 kBq). Longitudinal follow-up was performed over 1 wk, and 5 organs were studied: kidneys, lungs, heart, liver, and bones (Figure 2A). Antibody uptake was lowest in the lungs (AUC_0to168_: 531 ± 28 kBq.h/ml) followed by kidneys (AUC_0to168_: 1,312 ± 100 kBq.h/ml), heart (AUC_0to168_: 1,528 ± 131 kBq.h/ml), and liver (AUC_0to168_: 3,714 ± 390 kBq.h/ml). The highest AUC_0to168_ (26,251 ± 2,501 kBq.h/ml) was found in the bone (located in the femoral epiphysis; Figure 2B).

#### *In vivo* PET-CT Biodistribution in Lung Cancer-Grafted Mice

Seven mice transplanted with tumour cells in the lung (left) were IV injected with [^89^Zr]DFO-anti-PDL1 (690 ± 33 kBq). As can be seen in the histological images (**Figures 4A,B**), PD-L1 is expressed in the tumoural cells.

**Figure 3:**
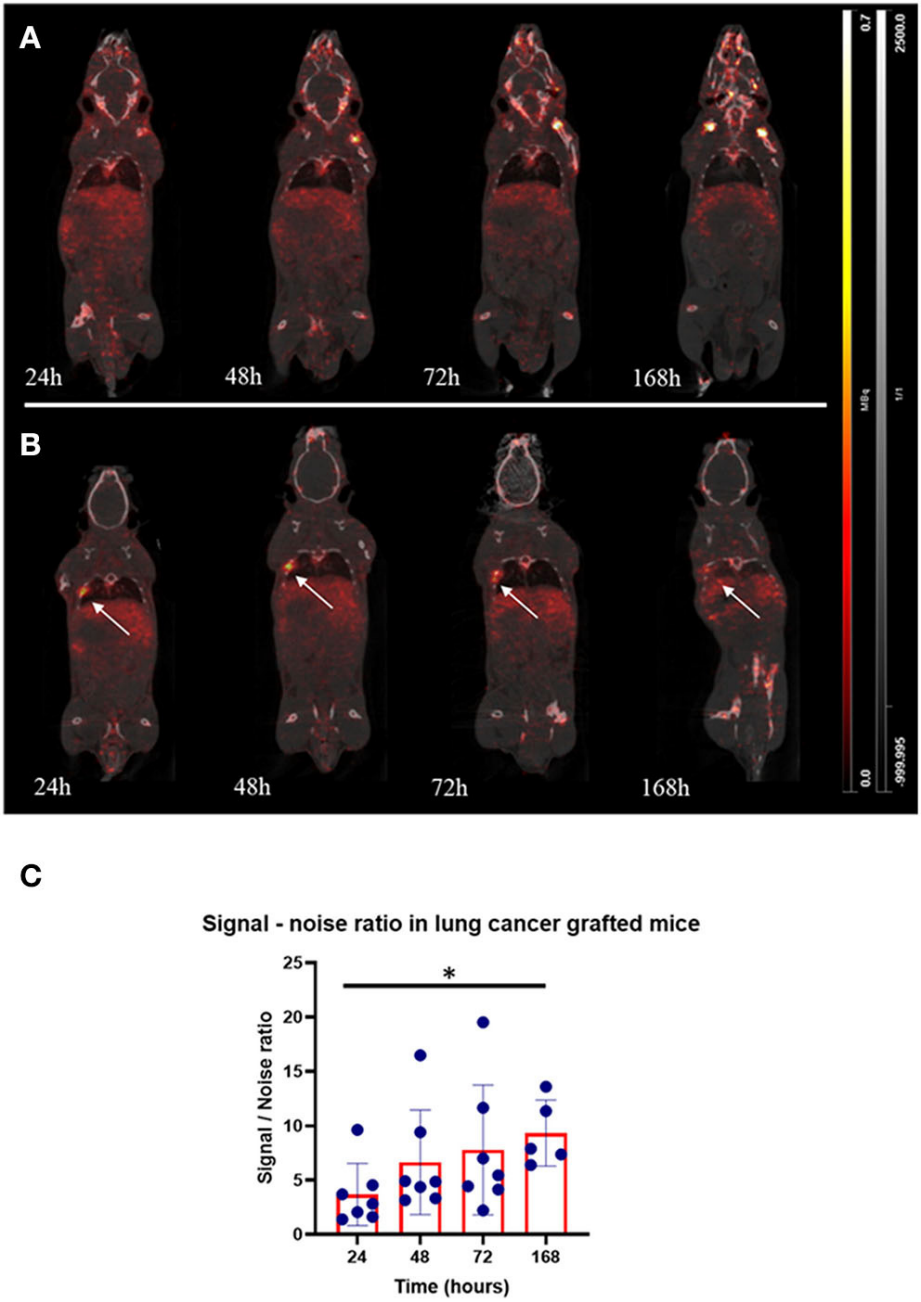
Preclinical imaging of [^89^Zr]DFO-anti-PDL1 in healthy and lung cancer-grafted mice. **(A)** Healthy mice after IV injection of [^89^Zr]DFO-anti-PDL1 at four times: 24, 48, 72, and 168 h. **(B)** Lung cancer-grafted mice after IV injection of [^89^Zr]DFO-anti-PDL1 at four times: 24, 48, 72, and 168 h. **(C)** Signal-to-noise ratio (SNR, tumour concentration/lung concentration). Kruskal-Wallis test (*p < 0.05).

The same time-activity curve profile was found in the five organs of interest for the grafted mice group compared to the healthy group. There were no significant differences in the AUC_0to168_ of organs, such as the heart (*p* = 0.22), lungs excluding the tumour injection site (*p* = 0.06), and kidneys (*p* = 0.68). However, bone AUC_0to168_ was greater in healthy mice (26,250 kBq.h/ml) than in transplanted mice (15,250 kBq.h/ml; *p* = 0.03), whereas liver AUC was higher in transplanted mice (*p* = 0.03; Figure 2B). The tumour signal was visible after 24 h in all transplanted mice (Figure 3B). The best signal-to-noise ratio (SNR, tumour to healthy lung within the same mice) was at 168 h (9.3 ± 3.0, CV was 36%; Figure 3C), however, there was no significant difference (*p* = 0.98) between this value and SNR at 48 h (6.6 ± 4.8, CV was 72%). The maximum tumour tracer concentration was found at 48 h after [^89^Zr]DFO-anti-PDL1 injection (41 ± 29 kBq/ml).

#### *Ex vivo* Biodistribution and Blocking Study

**Figure 4:**
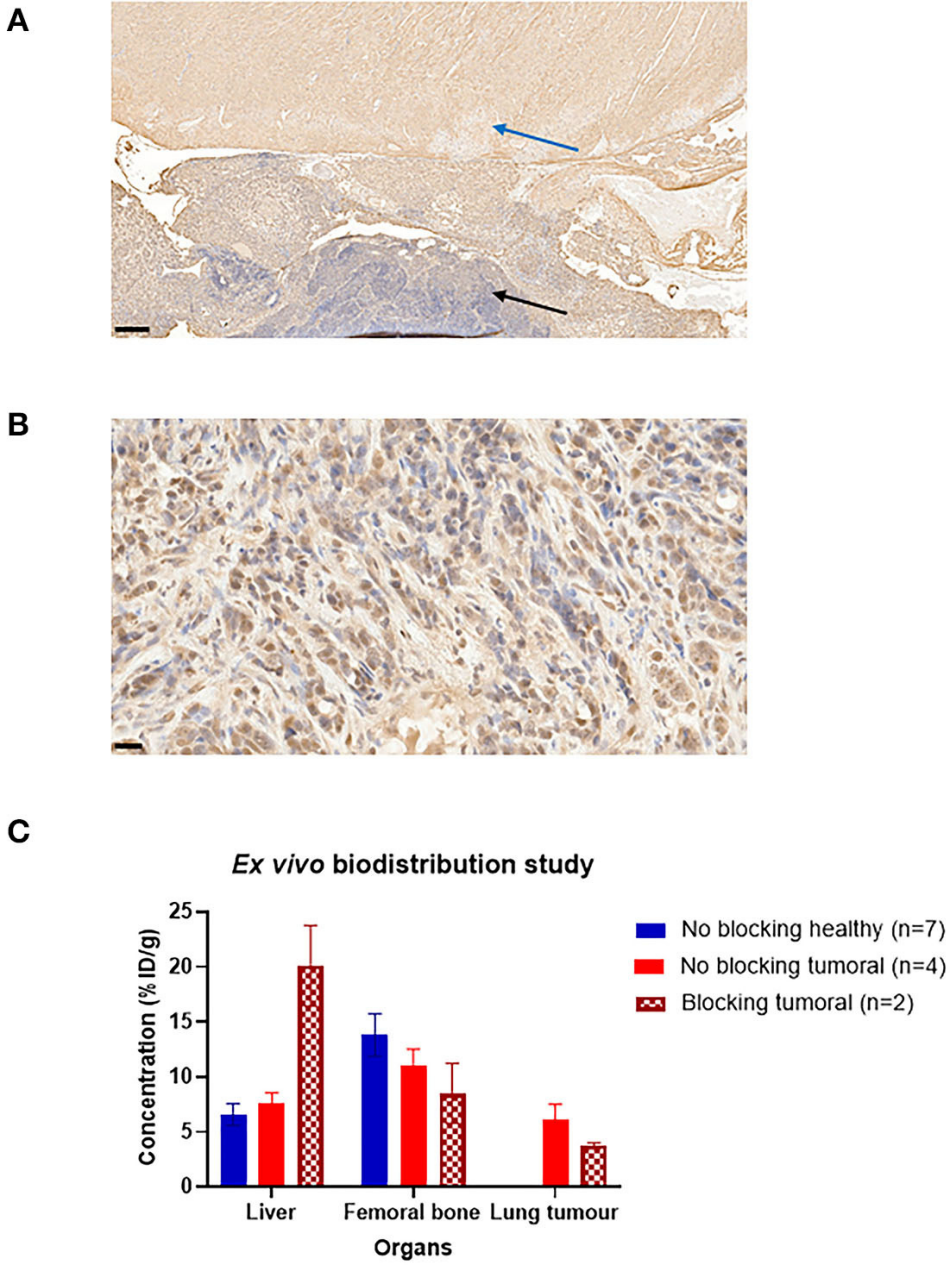
Histological expression of PD-L1 and *ex vivo* biodistribution study. **(A)** Histological slide (X5) of lung cancer-grafted mice 10 day after tumour induction. Blue arrows indicate the healthy lung tissue, black arrows indicate the tumoural tissue, black bar indicate the scale bar (200 μm). **(B)** On the same histological slide (X40), expression of PD-L1 is revealed by DAB around tumoural cells, black bar indicate the scale bar (20 μm). **(C)** *Ex vivo* biodistribution study in 3 groups: blocking tumoural group (co-injection of 500 μg of anti-PDL1), non-blocking healthy, and tumoural group.

*Ex vivo* studies were performed on 3 groups injected with [^89^Zr]DFO-anti-PDL1. Figure 4C shows the concentration of the [^89^Zr]DFO-anti-PDL1 in the liver, the femoral bone, and the lung tumour. When co-injected with 500 μg excess of anti-PDL1, tumoural concentration showed an almost 2-fold decrease (from 6.12 to 3.75%ID/g). However, liver concentration increased 3-fold in comparison to the non-blocking study (healthy or tumoural mice group) and bone concentration decreased from 11 to 8.5ID/g (in the tumoural group without blocking and blocking study, respectively).

#### PK Analysis of [^89^Zr]DFO-Anti-PDL1 in Blood

##### NCA

**Figure 5:**
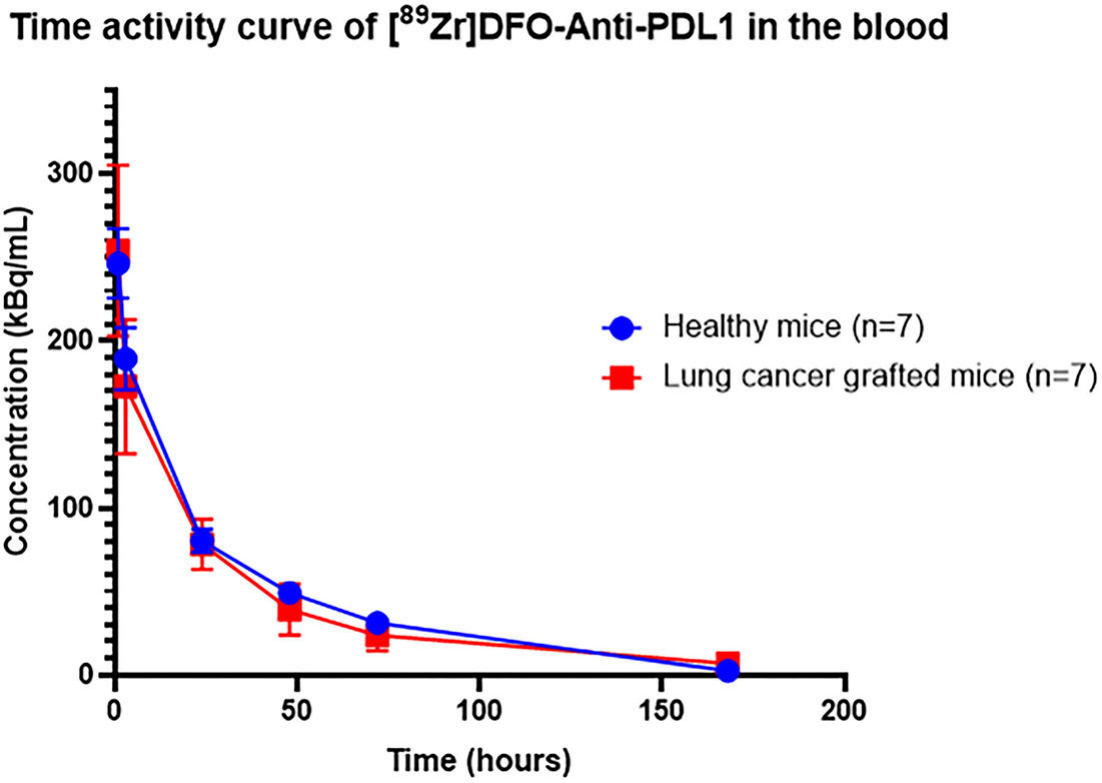
Time activity curve of [^89^Zr]DFO-anti-PDL1 in both healthy mice and lung cancer-grafted mice. Concentration values are decay corrected.

There was no difference in the injected activity between the tumoural group and the healthy group. Mean PK parameters were also similar, with no significant difference between groups for AUC, clearance, the volume of distribution, and the MRT (Table 1). However, the biological half-life was significantly different in healthy and grafted mice, with 28.7 h ± 1 and 38.5 h ± 10 (*p* = 0.03), respectively. Also, PK parameters in the tumoural group were more variable than in the healthy group (Table 1). The blood curve profile (Figure 5) is similar for both groups with a rapid distribution phase from the first hours and a slower elimination phase reflecting a bi-compartmental model. There was also greater variability in the concentration profiles of the lung cancer-grafted mice compared to the healthy mice.

**Table 1:** Blood PK parameters of healthy and lung cancer-grafted mice (NCA) estimated with the trapezoidal method.

**PK parameters**	**Healthy mice (*n =* 7) (CV%)**	**Lung cancer grafted mice (*n =* 7) (CV%)**	***P*-values (Wilcoxon)**
Activity (kBq)	742.3 (5.1%)	689.9 (4.7%)	0.07
AUC (kBq.h/mL)	6935.9 (4.8%)	6551.4 (13.2%)	0.71
Cl (mL/h)	0.11 (6.5%)	0.10 (20.9%)	0.09
Biological *T*_1/2_ (h)	28.7 (4.0%)	38.5 (26.6%)	0.03
MRT (h)	36.7 (2.8%)	37.9 (10.0%)	0.46
Vd (mL)	4.4 (5.6%)	5.6 (31.1%)	0.71

*AUC, the area under the curve; Cl, clearance; T_1/2_, half-life time; MRT, mean residence time; Vd, volume of distribution; CV(%), coefficient of variation; PK, pharmacokinetics*.

##### CA

The parameters of the 2-CMT model describe a bolus administration, first-order elimination (Cl), central compartment volume (V1) of distribution, peripheral compartment volume (V2) of distribution, and inter-compartmental clearance (Q). The parameters of the model are given in Supplementary Data.

**Figure 6:**
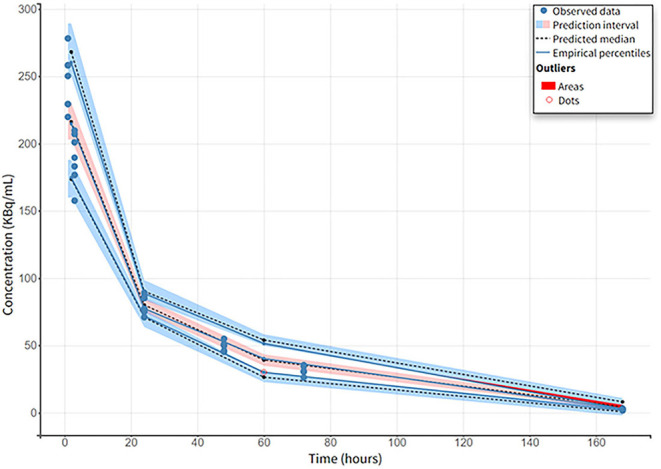
Visual predictive check of 2-CMT model of experimental blood data in healthy mice.

The 2-CMT model with the constant error described the experimental well data according to the Visual Predictive Cheque (VPC) (Figure 6) and individual concentration prediction. Parameters are estimated with good precision according to RSE and low parameter shrinkage (<10%). We compared the model-estimated AUC to the NCA-calculated AUC and found no significant differences (*p* = 0.53). Goodness-of-fit plots and VPC show a satisfactory fit of the model to the data. In addition, the error model does not show misspecifications according to the 90% prediction interval (Figure 6).

#### Healthy Mice Organ Dosimetry of [^89^Zr]DFO-Anti-PDL1 and Extrapolated Effective Dose

We used a 1-CMT CA model with an uptake constant (ka), a first-order elimination constant (ke), and a volume of distribution (V) to describe the distribution kinetics of the tracer at the organ level in mice and compared it with NCA AUC_0to168h_ estimation to validate its use for dosimetry calculation. The PK parameters were estimated with good precision (R.S.E <20%) and are given in Supplementary Data. The 1-CMT CA model was validated by calculating the RMSE between the CA AUC_0to168h_ estimate and the NCA AUC_0to168h_ estimate (Table 2). The 1-CMT CA model correctly estimates AUC_0to168h_ and will therefore be used to estimate AUC_0to infinity_.

**Table 2:** Comparison between NCA and CA mean AUC estimations.

**Organs**	**NCA AUC_**0to168h**_ (kBq.h/mL)**	**CA AUC_**0to168h**_ (kBq.h/mL)**	**RMSE (%)**
Heart	984	1,160	15
Lung	321	365	12
Kidney	756	837	11
Liver	1,899	2,026	8
Bone	12,353	12,619	6

*AUC, the area under the curve; relative RMSE, root mean square error relative to the model (NCA) estimation*.

Human dosimetry was estimated by total organ exposure to infinity (AUC_0toinfinity_) using PK modelling (Supplementary Data). Estimated absorbed organ dose was performed using IDAC-Dose 2.1 in normal tissues, based on the mice AUC_0toinfinity_ of [^89^Zr]DFO-anti-PDL1 (Table 3). The highest radiation exposure was found in the liver and in the gallbladder. According to ICRP 103, the effective dose was 131 μSv/MBq (±2.76%).

**Table 3:** Human extrapolated dosimetry with [^89^Zr]DFO-anti-PDL1 (IDAC-Dose) based on 7 healthy mice.

**Organs [mGy/MBq]**	**Adult male**	**CV%**
Adrenals	1.57E-01	4.81
Brain	8.99E-02	1.50
Breast	8.56E-02	1.76
Colon wall	1.02E-01	0.67
Endosteum (bone surface)	1.11E-01	0.73
ET region	8.18E-02	0.78
Eye lenses	6.24E-02	0.94
Gallbladder wall	2.07E-01	9.92
Heart wall	1.48E-01	5.61
Kidneys	1.44E-01	4.60
Liver	2.39E-01	12.3
Lung	1.51E-01	6.87
Lymphatic nodes	1.10E-01	0.69
Muscle	9.39E-02	1.56
Oesophagus	1.31E-01	3.25
Oral mucosa	9.38E-02	1.14
Pancreas	1.50E-01	4.87
Prostate	1.14E-01	2.75
Red (active) bone marrow	1.32E-01	0.92
Salivary glands	8.65E-02	1.26
Skin	6.55E-02	1.73
Small intestine wall	1.03E-01	1.22
Spleen	1.07E-01	0.73
Stomach wall	1.18E-01	3.84
Testes	8.77E-02	3.87
Thymus	1.11E-01	1.64
Thyroid	1.00E-01	0.71
Urinary bladder wall	1.05E-01	2.64
Effective dose 103 [mSv/MBq]	1.31E-01	2.76

*Sv, Sievert; Bq, Becquerel; Gy, gray; CV%, coefficient of variation*.

## Discussion

The aim of our study was to evaluate the biodistribution of [^89^Zr]DFO-anti-PDL1 in healthy and lung cancer-grafted immunocompetent mice and estimate human dosimetry by extrapolating the PK parameters in organs from mouse to human.

### *In vitro* Validation

An *in vitro* step was crucial to assess the potential damage of the antibodies after the radiolabelling. An IR of 96% was found indicating that the antibodies were not damaged by our radiolabelling process. Kikuchi et al. (36) radiolabelled the same clone (10F.9G2) with [^89^Zr] in different conditions (*p*-NCS-DFO ratio of 5:1 and 4°C incubation vs. a *p*-NCS-DFO ratio of 3:1 and 37°C incubation in our study) and found IRs between 55 and 70%. A second experiment was carried out to estimate the affinity of [^89^Zr]DFO-anti-PDL1 to its target (Kd = 5.6 nM). The affinity was in line with the binding constants found for antibodies in the nanomolar range (37).

We were not able to find other studies to which our results regarding the affinity binding (clone 10F.9G2) could be compared. When tested in PSB, the [^89^Zr]DFO-anti-PDL1 had an RCP >95% on day 7 whereas RCP was only 75% in the plasma. Therefore, we can consider that plasma has an influence on the stability of the radiolabelled antibody. Even though this *in vitro* study in plasma is not strictly representative of the *in vivo* behaviour of the radiolabelled antibody because of the remaining blood activity in mice on day 7, we tried to find an alternative method to investigate possible enzymatic plasma degradation and the influence of the physicochemical plasma condition. Indeed, on day 7, the remaining blood activity in mice was about 2.3kBq/ml which is too low to be detected by a gamma detector paired with HPLC and therefore too low to perform an *in vivo* stability study.

Concerning the degradation mechanism, Vugts et al. (38) assessed the stability of different [^89^Zr] chelators in the plasma. Changing the chelator DFO (used in our study) by DFO^*^ resulted in a radiochemical purity >95% in the plasma (and RCP of 75% while using DFO). Furthermore, when Vugts et al. tested the stability of the DFO with the NaCl 0.9%, they found an RCP of 50.4% on day 7. Based on these results, we suspect that [^89^Zr] is released from the chelator due to the physicochemical condition and not because of enzymatic metabolism.

### Biodistribution of [^89^Zr]DFO-Anti-PDL1 in Healthy Mice

The concentrations of [^89^Zr]DFO-anti-PDL1 found in the lung presented the lowest AUC_0to168h_ (321 kBq.h/ml) reflecting a weak background noise on PET imaging. Organs, such as the heart, the kidneys, and the liver, had between 4- and 10-fold higher AUC_0to168h_ explained by their richer vascularization.

High liver concentration over time could describe a non-specific clearance (39–41). A non-specific clearance occurs through proteolytic degradation and pinocytosis leading to the degradation of the antibody into amino acids or peptides (42). These peptides could contain [^89^Zr] and accumulate within the liver.

Renal elimination through glomerular filtration (cut-off 30–50 kDa) is insignificant due to high molecular weight (*MW*_*IgG*_ 150 kDa). Kidney signal is likely to correspond to free [^89^Zr] and/or [^89^Zr]DFO since antibodies are neither filtered nor secreted at the nephron level (43, 44). One major limitation of our study is the absence of radiometabolism analysis in the blood and the organs. However, there are studies that explore the behaviour of [^89^Zr]-labelled antibodies and their radiometabolites. Abou et al. (45) investigated biodistribution of [^89^Zr]DFO and [^89^Zr]Phosphate in healthy mice. They noticed that [^89^Zr]DFO was totally cleared from the body after 1 day but [^89^Zr]Phosphate exhibited similar kidney and liver concentration levels as those found in our healthy mice. Therefore, the remaining concentration found in the liver and the kidneys is likely to be a product of the metabolization of the radiolabelled antibodies, such as [^89^Zr]Phosphate. Holland et al. (46) also investigated the biodistribution of the [^89^Zr]DFO in mice and revealed that after 1 min, the [^89^Zr]DFO was mainly located in the kidney and after 4 min, the [^89^Zr]DFO was majorly located in the bladder confirming the high renal clearance of the [^89^Zr]DFO.

One of the major issues using the [^89^Zr]-radiolabelled antibodies is the release of free [^89^Zr]. The free [^89^Zr] will accumulate in bones (e.g., epiphysis). The structure of DFO provides 6 coordination sites while 8 sites are required to form a stable complex, this can result in the release of [^89^Zr], which may subsequently accumulate in mineral bone (45–49). In our study, the region of interest of the bones is positioned at the level of the femoral epiphysis. This organ had the highest AUC_0to168h_ (on average, 10-fold higher than in the heart or kidney). At this age (6–8 wk in our experiment) mice are still growing. As explained by Ferguson et al. (50), the percentage of mineralization increases at the femoral epiphysis location, participating in bone remodelling and bone growth in mice, which explains the affinity of free [^89^Zr] for the bone.

In comparison, the remaining bone activity at 168 h was around 24% of injected activity per gramme (%ID/g) whereas Li et al. (51) found around 10%ID/g in the bone (mice aged 4–5 wk). As our mice are immunocompetent, they could have a higher non-specific clearance resulting in the more proteolytic activity of the antibody, which in turn could lead to more radiolabelled metabolite and higher bone uptake (39–41).

### Biodistribution of [^89^Zr]DFO-Anti-PDL1 in Lung Cancer-Grafted Mice

The distribution of the tracer in transplanted mice followed the same progression as in healthy mice with respect to the following organs: bones, heart, liver, kidneys, and lungs. The tumour uptake was visible 24 h after the injection, and the maximum average concentration of [^89^Zr]DFO-anti-PDL1 was reached after 48 h. We were able to successfully target the tumour within 24 h after injection and demonstrated that [^89^Zr]DFO-anti-PDL1 can be used for the non-invasive imaging of the CMT167 lung cancer tumour in a syngeneic mice model.

As demonstrated in the blocking study, adding 500 μg of cold anti-PDL1 halved the tumoural signal due to a competitive interaction between cold anti-PDL1 and radiolabelled anti-PDL1 on the tumoural cells. Other elements in our study support specific binding of anti-PDL1. For example, in the PK analysis, the half-life of [^89^Zr]DFO-anti-PDL1 was significantly higher in the LCG mice (38.5 h) compared to the healthy group (28 h), which seems to indicate that the difference could be due to the targeting of the tumour. These two results support the hypothesis that specific binding does occur. *In vitro* and *in vivo* results by Li et al. (29) also showed that after 1 wk of tumoural development, PD-L1 is expressed in the same murine lung.

In the blocking study, liver concentration increases 3-fold in comparison to the non-blocking study. PD-L1 is ubiquitously expressed in the body (e.g., spleen, liver, or the bone marrow) (52). With more than 500 μg (>20 mg/kg), specific sites could be saturated by the excess cold in favour of non-specific clearance conducted by the liver (53).

In contrast, femoral bone showed a lower uptake in the blocking group and could probably indicate PD-L1 expression at the femoral level. As described by Li et al. (29), bone marrow-derived macrophage tends to express PD-L1 in the case of CMT167 lung cancer-induced mice. In another study published by Wang et al. (54), authors assessed the sensitivity of the PD-1 treatment (nivolumab) on CMT167-induced cancer (bone cancer associated), and PD-L1 was expressed at baseline (exosome serum). These findings suggest that a portion of bone uptake is related to the possible bone PD-L1 expression.

According to radioactive signal and SNR, the best imaging time is 48 h after injection. No significant differences in tumoural uptake were found between 24 and 168 h indicating that the tumoural signal could be due to the specific targeting of the [^89^Zr]DFO-anti-PDL1 and its internalisation. Depending on the antibody internalisation, [^89^Zr] remains trapped inside the cell after antibody internalisation and degradation leading to an accumulation over time of the signal in the tumour or targeted cell (21, 55). In 2020, Kurino et al. (56) performed the biodistribution of the 10F.9G2, which is the same clone as in our study. They investigated the biodistribution and the radiometabolism of the antibody using Iodine-125 (covalent binding) and Indium-111 (radiolabelling by chelation as it is for [^89^Zr]). They found a high rate of degradation linked to the expression of PD-L1 in organs, such as the spleen, liver, and tumour. For Iodine-125, the radioisotope leaves the cell after antibody internalisation and degradation whereas for Indium-111, the radioisotope stays in the cell after antibody internalisation and degradation. Despite the clear limitation in the comparison (tumoural model, the dose, and the radiolabelling method), Kurino et al. supported the underlying PK and metabolism of [^89^Zr]DFO-anti-PDL1.

Other interactions, such as enhanced permeability and retention effect (EPR), are known to drag macromolecules in the tumoural compartment, which could lead to the retention of the tumoural signal and participate in the non-specific signal (57, 58).

### PK and Dosimetry of [^89^Zr]DFO-Anti-PDL1

PET imaging offers the possibility to assess the distribution of the studied molecule in various organs. Compared to standard preclinical experiments, access to imaging reduces the number of mice necessary and enables longitudinal data to be collected. The blood time-activity curves of the tracer in healthy and lung cancer-grafted mice were similar. They describe a rapid distribution of the tracer followed by significant elimination. However, we found greater variability in the PK parameters within the tumoural group (Table 1). This variability could be due to the induced tumour and may affect the overall tumoural kinetics of [^89^Zr]DFO-anti-PDL1. Depending on the expression of PD-L1, size of the tumour, and injection site, [^89^Zr]DFO-anti-PDL1 uptake could vary between subjects and may impact the volume of distribution.

The final blood PK model in healthy mice is a 2-CMT model, and it concurs with other PK models for antibodies (59). But, with a few individuals (*n* = 7), it is difficult to correctly estimate all parameters (such as IIV). In our case, very low variability was visible in the dataset facilitating the overall estimation of the parameters (Figures 5, 6).

We also used a 1-CMT model to calculate the AUC in the organs for a dosimetry assessment. We were able to estimate the PK parameters with good precision (RSE <20%), and RMSE was below 20% for AUC_0to168h_ estimation. Therefore, we used this 1-CMT model to estimate AUC _infinite_ to calculate organ AUC in mice and extrapolate the PK in human. Allometric scale factors between human and mice are not constant between organs according to the McParland equation. For instance, kidney and liver have 2.3 and 1.4, respectively, compared to bone and lung with 0.5 and 0.4, respectively. Therefore, this could explain the low radiation exposure in some organs (e.g., bone) even though their estimated AUC is high (Supplementary Data).

Despite some limitations (differences between species in biotransformation, non-linear PK, or alteration of physiological pathways), allometric scaling has been shown to be a reliable method to predict the main PK parameters (such as the clearance or the volume of distribution) in the human based on animal data (26). In some cases, the dosimetry predicted from mice can be overestimated, such as in the study by Bhattacharyya et al. (60), where an effective dose of 578 μSv/MBq was found for the [^89^Zr]DFO-panitumumab but was lower (264 μSv/MBq) in the clinical investigation published by Lindenberg et al. (61).

The extrapolated effective dose was 131 μSv/MBq. In comparison, [^18^F]FDG has an effective dose around 20 μSv/MBq (62). Despite this difference, the effective dose was lower than expected in view of the gamma energy of [^89^Zr] (*E* = 910 keV) and existing literature. Indeed, Jagoda et al. (37) performed a dosimetry evaluation in humans from experimental mice biodistribution using avelumab (anti-PDL1) and found an effective dose of 363 μSv/MBq. The difference between our results and theirs could be explained by the respective *in vivo* models and specific activities. We used a fully immunocompetent lung cancer-grafted model whereas Jagoda et al. (37) used athymic mice (subcutaneous breast cancer model). Also, with lower specific activity in our case, we have a competition between non-radiolabelled and radiolabelled antibodies leading to a faster elimination of our [^89^Zr]DFO-anti-PDL1.

## Conclusion

In this study, we were able to successfully radiolabel an anti-PDL1 with [^89^Zr] with no damage according to the IR. The PK and biodistribution were explored in both healthy mice and lung cancer-grafted immunocompetent mice. The best time for tumour imaging was 48 h. Human radiation exposure was estimated using PK modelling with a human effective dose of 131 μSv/MBq. Radiolabelled antibodies could improve personalised medicine for PD-1/PD-L1 treatment by targeting tumoural PD-L1 expression. Data shared here could help further research to support the clinical development of this diagnostic biomarker.

## Data Availability Statement

The raw data supporting the conclusions of this article will be made available by the authors upon request to anis.krache@inserm.fr.

## Ethics Statement

The animal study was reviewed and approved by French Ethics Committee (No. 22816-2019111216307851).

## Author Contributions

A-SS, CF, and MW-K designed the project. AK, CF, and A-SS contributed to the labelling process. CP contributed to the imaging acquisition. MB contributed to dosimetry calculation. AK was responsible for data analysis, imaging analysis, data interpretation, and wrote the manuscript. CF, A-SS, MW-K, EC, and PP revised the manuscript. All authors have read and approved the final manuscript.

## Funding

This study was funded by the European Regional Development Fund (Grant No. 16015075/MP0014063) and Occitania Region (Mutualised Platform Grant No. 16015090) PIR^2^ and Labex IRON (11-LABX-0018).

## Conflict of Interest

Employment: CF and YB are employees of General Electric-Zionexa laboratory. The remaining authors declare that the research was conducted in the absence of any commercial or financial relationships that could be construed as a potential conflict of interest.

## Publisher's Note

All claims expressed in this article are solely those of the authors and do not necessarily represent those of their affiliated organizations, or those of the publisher, the editors and the reviewers. Any product that may be evaluated in this article, or claim that may be made by its manufacturer, is not guaranteed or endorsed by the publisher.
