# Supplementary material for: Preclinical Pharmacokinetics and Dosimetry of an 89Zr Labelled Anti-PDL1 in an Orthotopic Lung Cancer Murine Model
Source: Front Med (Lausanne). 2022 Jan 31;8:741855. doi: 10.3389/fmed.2021.741855 (PMC8841431; doi:10.3389/fmed.2021.741855)
Supplement: Supplementary file 1 [file Data_Sheet_1.pdf]

Mice dosimetry estimation and human absorbed radiation estimation

Table 1: McParland allometric factor estimation

Organs	Mean organ human [male] weight (g)	Mean organ mice [male] weight (g)	Ratio [mice/human] Whole body (g/g)	Ratio [human/mice] organ (g/g)	Allometric scale factor
**Heart**	386	0.137	0.00035	2817	0.986
**Bone (Endosteum)**	580	0.096	0.00035	6041	2.114
**Liver**	2360	1.115	0.00035	2166	0.741
**Kidney**	422	0.334	0.00035	1263	0.442
**Lung**	1200	0.144	0.00035	8333	2.917
**The rest of the body**	65052	23.14	0.00035	2811	1.004

For an average male (70 kg) and average mice (25g)

Table 2: Human absorbed radiation estimation

Organs	Mean AUC $_{infinite}$ in mice (kBq.h/mL)	Organ weight (g)	Tissue density (g/mL)	Mean AUC $_{infinite}$ in mice (kBq.h/g)	Cumulated activity (kBq.h)	Mean residence time in mice (h)	Allometric scale factor	Human estimated activity (kBq.h)	Mean residence time in human (h)
**Heart**	1165.9	0.137	1.04	1121	153.8	0.21	1.036	160.3	0.204
**Bone (Endosteum)**	15510.3	0.096	1.4	11079	1068.9	1.44	0.484	526.5	3.046
**Liver**	2422.4	1.115	1.04	2329	2592.4	3.49	1.379	3678.3	2.588
**Kidney**	861.8	0.334	1.04	829	277.3	0.37	2.316	647.8	0.165
**Lung**	371.1	0.144	0.29	1254	181.9	0.26	0.351	65.9	0.715
**The whole-body**	1063	25	1	1063	26575	33	-	-	-
**The rest of the body**	-	-	1	-	-	27.23	1.004	-	27.28

Mean AUC $_{infinite}$ in mice and organ weight are experimental data. The rest of the body MRT was calculated by subtracting the MRT in five organs from the whole-body MRT

Table 3: Pharmacokinetic parameters of 1-CMT model in mice for AUC extrapolation to infinity

Organs	Ka (h^{-1})	RSE (%)	Ke (h^{-1})	RSE (%)	V (mL)	RSE (%)
**Heart**	0.028	6.4	0.157	14.9	2.2	18
**Bone**	0.0127	12.7	0.0188	4.6	1.84	8.3
**Liver**	0.0103	10.2	0.124	11.8	1.26	7.2
**Kidney**	0.0183	10.6	0.179	6.1	2.44	9.3
**Lung**	0.0228	14.2	0.155	7.7	6.91	7

Table 4: Blood PK parameters of healthy mice (CA) estimated from the 2-CMT model.

Parameter	VALUE	Units	R.S.E (%)	Shrinkage (%)
**Clearance**	0.1	mL/h	3.2	3.8
**IIV of Clearance**	7.4	CV%	32.4	-
**Volume of central compartment (V1)**	2.6	mL	3.3	2.8
**IIV of V1**	8.3	CV%	29.1	-
**Inter-compartment clearance (Q)**	0.3	mL/h	12.1	7.6
**IIV of Q**	30	CV%	29.8	-
**Volume of peripheral compartment (V2)**	2.3	mL	6.4	8.9
**IIV of V2**	12	CV%	50.6	-
**Error model a**	2.8	kBq/mL	18.7	-
**AUC model-based**	6795	kBq.h/mL	-	-
**IIV of AUC model based**	5.7	CV%		-

IIV: Inter-individual variability, error model a: constant model error, RSE: relative standard error

PET method validation

5 VOI per organ were drawn according to the visual contouring under PET-CT image for the partial method (Figure S1). We compared this method to the whole organ contouring (Figure S1). To validate the method, we compared partial method concentration extracted from the liver and heart of healthy mice (n=3) to the whole organ contouring (Table 5) at day 1. The difference between both methods were statistically not different (P = 0.68). Therefore, the partial method offers a suitable surrogate to quantify the concentration in the organs.

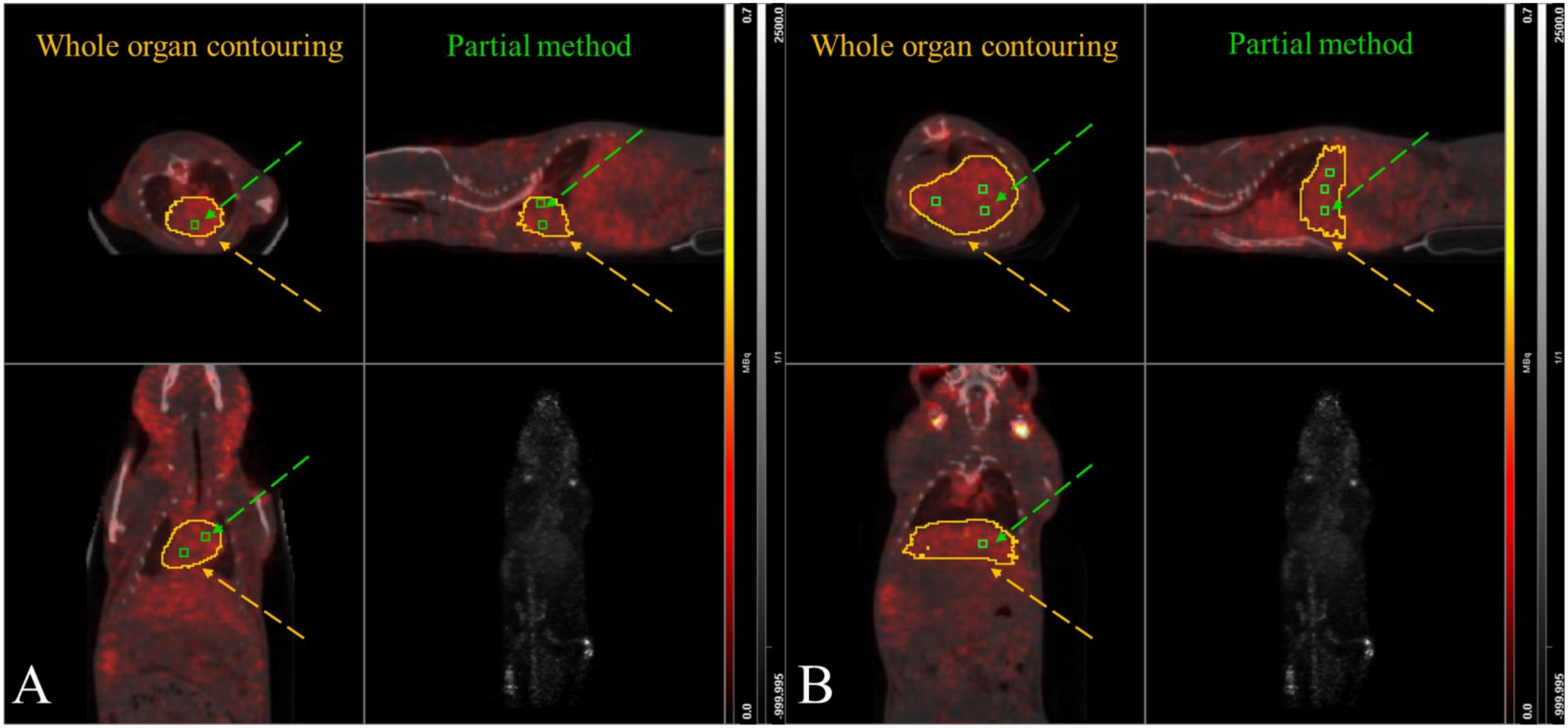

Figure S1: Organ segmentation and concentration extracted from the partial method. (A) heart concentration was estimated using whole organ contouring VOI (orange arrow) and partial method VOI (green arrow). (B) liver VOI representation.

Table 5: Comparison between the partial method VOI versus the whole method organ contouring

		**Partial method**						**whole organ method**
**Organ**	Mice	VOI1	VOI2	VOI3	VOI4	VOI5	**MEAN (CV%)**	**Full VOI**
**Unit**					MBq/mL			
**Heart**	ID 1	0.11	0.13	0.11	0.11	0.11	**0.12 (±5.6)**	**0.092**
	ID 2	0.07	0.07	0.09	0.08	0.09	**0.08 (±10)**	**0.090**
	ID 3	0.07	0.08	0.08	0.07	0.10	**0.08 (±10)**	**0.091**
**Liver**	ID 1	0.079	0.101	0.116	0.111	0.108	**0.10 (±10)**	**0.091**
	ID 2	0.104	0.085	0.097	0.078	0.083	**0.089 (±9.9)**	**0.083**
	ID 3	0.108	0.086	0.095	0.097	0.108	**0.099 (±7.4)**	**0.091**

Table 5 provide an overview of the radioactive concentration calculation within the organ using the partial method and whole method contouring. CV: Coefficient of variation

Table 6: Concentration comparison between whole method contouring and partial method

Organ	Liver		Heart	
**Method**	**Whole method contouring**	**Partial method**	**Whole method contouring**	**Partial method**
**Values (MBq/mL)**	34.15	38.22	33.22	35.24
	30.21	34.27	32.86	32.56
	34.21	37.49	34.34	37.04
	30.79	32.44	30.13	31.18
	36.31	29.79	32.05	33.12
	30.59	32.62	24.32	22.54
**P values (Wilcoxon-test)**	**0.48**		**0.86**	

Table 6 compares extracted concentration from the partial method and the whole method contouring at day 1 and for 6 distinct mice. Non-parametric Wilcoxon-test was used to compare the average concentration extracted from both method and no statistical differences were found for the heart and the liver.

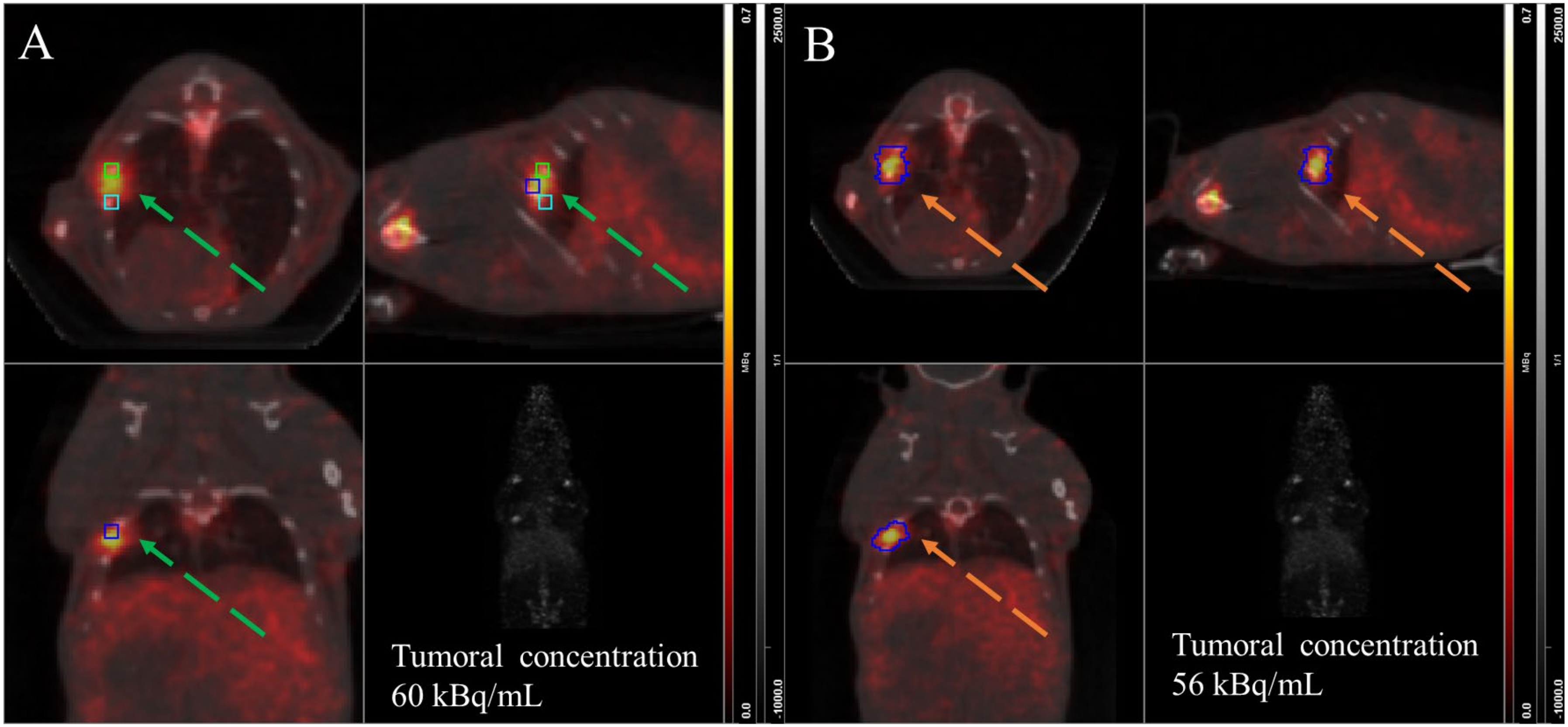

Figure S2: Partial method and whole tumour contouring at day 2. (A) shows a PET-CT image of lung grafted mice. An average concentration of 60 kBq/mL (± 4%) was estimated using partial method (green arrow). (B) whole tumoral contouring (orange arrow) with an estimated concentration of 56 kBq/mL.

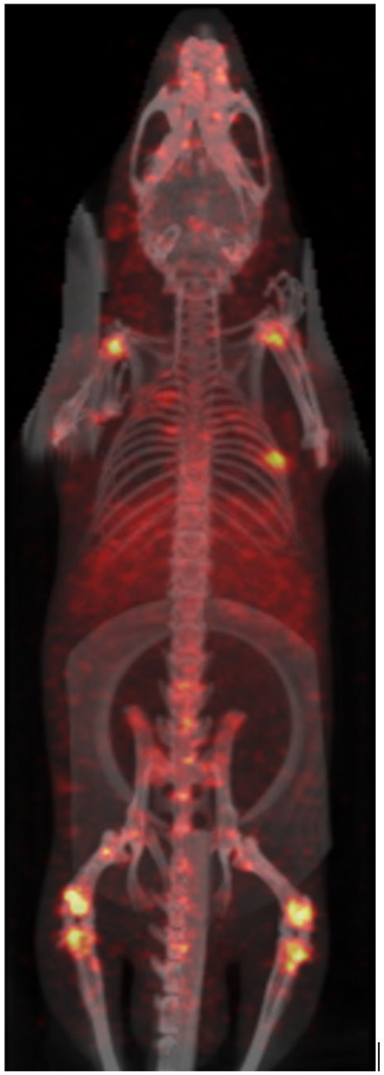

Figure S3: Tumoral biodistribution of [^{89}Zr]DFO-Anti-PDL1 2 days after I.V caudal injection (0.7 MBq). Maximal Intensity projection (MIP) static PET image was generated using Mixing fusion mode.

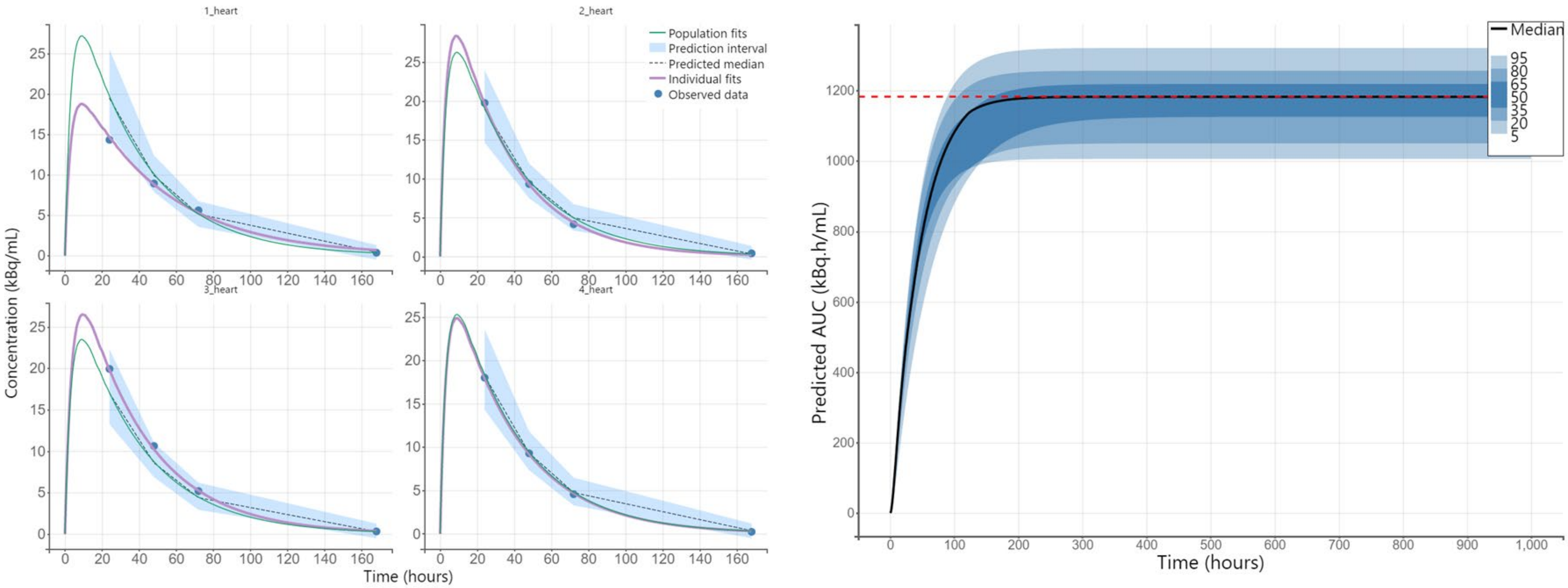

Figure S4: POP_PK curve fitting using 1-CMT model for dosimetry and AUC estimation. The experimental data (blue dots) are correctly captured by the PK model (purple line). Thus, the AUC is estimated using the PK parameters of the corresponding model.
